# QuickStats: Percentage* of Women Aged ≥50 Years Who Have Had a Hysterectomy,^†^ by Race/Ethnicity and Year — National Health Interview Survey, United States, 2008 and 2018^§^

**DOI:** 10.15585/mmwr.mm6841a3

**Published:** 2019-10-18

**Figure:**
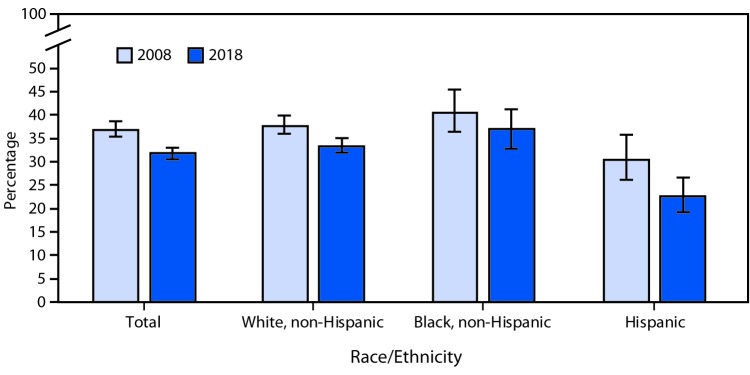
The percentage of women aged ≥50 years who have had a hysterectomy decreased from 36.6% in 2008 to 31.7% in 2018. Decreases were also observed among non-Hispanic white women (37.5% to 33.3%) and Hispanic women (30.3% to 22.6%), but there was no significant decrease for non-Hispanic black women (40.4% to 36.8%). For both time points, non-Hispanic black and non-Hispanic white women were more likely than Hispanic women to have had a hysterectomy.

**Source:** National Health Interview Survey, 2008 and 2018 data. https://www.cdc.gov/nchs/nhis.htm.

